# Supplementary material for: Non-communicable diseases risk factors and their determinants: A cross-sectional state-wide STEPS survey, Haryana, North India
Source: PLoS One. 2019 Nov 27;14(11):e0208872. doi: 10.1371/journal.pone.0208872 (PMC6881003; doi:10.1371/journal.pone.0208872)
Supplement: S1 Table — (DOCX) [file pone.0208872.s001.docx]

**Definitions used**

Table 1: Cut-off Criteria used in the Survey

| S.No. | Variable/Indicator | Cut-off |
| --- | --- | --- |
| 1. | At Risk for NCDs | <5 servings of fruit/vegetables |
| 2 | Overweight | 25-29.9 kg/m^2^ |
| 3 | Obesity | >30 kg/m^2^ |
| 4 | Abdominal obesity | ≥90 cm in men  ≥80 cm in women |
| 5 | Hypertension | systolic blood pressure of ≥140 mm of Hg  or a diastolic blood pressure of ≥90 mm of Hg  or/and use of anti-hypertensive medications |
| 6 | Diabetes mellitus | fasting plasma glucose of ≥110 mg /dl  OR  on medications for high blood sugar |
| 7. | Hypercholesterolemia | Total Cholesterol ≥190 mg/dl |
| 8. | Hypertriglyceridemia | Serum triglyceride ≥150 mg/dl |
| 9. | Raised intake of sodium | Salt intake > 5gm per day |
