# Supplementary material for: Non-communicable diseases risk factors and their determinants: A cross-sectional state-wide STEPS survey, Haryana, North India
Source: PLoS One. 2019 Nov 27;14(11):e0208872. doi: 10.1371/journal.pone.0208872 (PMC6881003; doi:10.1371/journal.pone.0208872)
Supplement: S1 File — (DOCX) [file pone.0208872.s002.docx]

**Data Collection**

Repeat for 3 times

Steps for approaching the participants

Day 1: Registration and interview for behavioral risk factors

Postpone Day 2, Appointment rescheduling with fasting instructions

Fasting? If Yes

Blood and Urine Samples from every 2^nd^ participant

Day 2: Assessment of participant’s fasting status

Anthropometric measurements with instructions for fasting

15 minutes rest and Blood Pressure measurements

Results oriented counseling and referral followed by checkout

If No
