# Supplementary material for: Non-communicable diseases risk factors and their determinants: A cross-sectional state-wide STEPS survey, Haryana, North India
Source: PLoS One. 2019 Nov 27;14(11):e0208872. doi: 10.1371/journal.pone.0208872 (PMC6881003; doi:10.1371/journal.pone.0208872)
Supplement: S2 File — (PDF) [file pone.0208872.s003.pdf]

NON- COMMUNICABLE DISEASE RISK FACTORS SURVEY IN HARYANA, INDIA
STEPS INSTRUMENT VERSION 3.1 (ADAPTED)

Survey Information		
**Location and Date**	**Response**	**Code**
**Unique ID (Cluster (CEB/Village) ID and Patient ID)**	__ ___ __ ___ ___	I1
**Cluster (CEB/Village) name**		I2
**Interviewer ID**	└─┴─┴─┘	I3
**Date of completion of the instrument**	└─┴─┘ └─┴─┘ └─┴─┴─┴─┘ **dd** **mm** **year**	I4

**Consent, Interview Language and Name**	**Response**	**Code**
Consent has been read and obtained	Yes 1<br>No 2 **If NO, END**	I5
Interview Language	English 1<br>*Hindi* 2<br>*Punjabi* 3<br>*Other* 4	I6
Time of interview<br>(24 hour clock)	└─┴─┘ : └─┴─┘<br>hrsmins	I7
Family Surname		I8
First Name		I9
Contact phone number where possible		I10
Unique Identification Number		I11

Demographic information			
**Question**	**Response**		**Code**
Sex (*Record Male / Female as observed)*	Male	1	C1
	Female	2	
What is your date of birth? *Don't Know 77 77 7777*	__|__ __|__ __|__|__|__ dd mm year	*If known, Go to C4*	C2
How old are you?	Years	__|__	C3
In total, how many years have you spent at school and in full-time study (excluding pre-school)?	Years	__|__	C4
What is **the highest level of education** you have completed?	No formal schooling	1	C5
	Less than primary school	2	
	Primary school completed	3	
	Secondary school completed	4	
	High school completed	5	
	College/University completed	6	
	Post graduate degree	7	
	Refused	88	
What is your *social group?*	SC	1	C6
	OBC/others	2	
	General	3	
	Refused	88	
What is your ethnic sub group?	Jaat	1	R 1E
	Yaduvanshi	2	
	Ahirs	3	
	Kambojis	4	
	Gujjars	5	
	Brahmins	6	
	Rajputs	7	
	Ror	8	
	Saini	9	
	Punjabi	10	
	Other (please specify):	------------------	
	Refused	88	
What is your **marital status**?	Never married	**1**	C7
	Currently married	2	
	Separated	3	
	Divorced	4	
	Widowed	5	
	Cohabitating	6	
	Refused	88	

Which of the following best describes your **mainwork** status over the past 12 months?<br>(USE SHOW CARDS)	Government employee	1	C8
	Non-government employee	2	
	Self-employed	3	
	Non-paid	4	
	Student	5	
	Homemaker	6	
	Retired	7	
	Unemployed (able to work)	8	
	Unemployed (unable to work)	9	
	Refused	88	
How many people older than 18 years, including yourself, live in your household?	Number of people	└─┴─┘	C9
Taking the past year, can you tell me what the average earnings of the household have been?<br>*(RECORD ONLY ONE, NOT ALL 3)*	per month	└─┴─┴─┴─┴─┴─┴─┘	C10a
	OR per year	└─┴─┴─┴─┴─┴─┴─┘	C10b
	Refused	88	C10c

Behavioural Measurements

Tobacco Use

Now I am going to ask you some questions about tobacco use.

Question	Response			Code
Do you **currently** smoke any **tobacco** products, such as bidis, cigarettes, cigars or pipes, hookah or any other local tobacco products?<br>(USE SHOW CARDS)	Yes	1		T1
	No	2	*If No, go to T8*	
Do you currently smoke tobacco products **daily**?	Yes	1		T2
	No	2		
How old were you when you **first started** smoking?	Age (years)<br>Don't know 77	└─┴─┘	*If Known, go to T5a/T5aw*	T3
Do you remember how long ago it was?<br>*(RECORD ONLY 1, NOT ALL 3)*<br>*Don't know 77*	In Years	└─┴─┘	*If Known, go to T5a/T5aw*	T4a
	OR in Months	└─┴─┘	*If Known, go to T5a/T5aw*	T4b
	OR in Weeks	└─┴─┘		T4c
On average, **how many** of the following products do you smoke **each day/week?**<br>*(IF LESS THAN DAILY, RECORD WEEKLY)*<br>*(RECORD FOR EACH TYPE, USE SHOWCARD)*<br>*Don't Know 7777*		DAILY↓	WEEKLY↓	
	Manufactured cigarettes	└─┴─┴─┴─┘	└─┴─┴─┴─┘	T5a/T5aw
	Hand-rolled cigarettes/ bidis	└─┴─┴─┴─┘	└─┴─┴─┴─┘	T5b/T5bw
	Pipes full of tobacco	└─┴─┴─┴─┘	└─┴─┴─┴─┘	T5c/T5cw
	Cigars, cheroots, cigarillos	└─┴─┴─┴─┘	└─┴─┴─┴─┘	T5d/T5dw
	Number of Shisha/hookah sessions	└─┴─┴─┴─┘	└─┴─┴─┴─┘	T5e/T5ew

	Other	└─┴─┴─┴─┘ └─┴─┴─┴─┘ *If Other, go to T5other, else go to T6*	T5f/T5fw
	Other (please specify):	└─┴─┴─┴─┴─┴─┘	T5other/ T5otherw
During the past 12 months, have you tried to **stop smoking**?	Yes<br>No	1<br>2	T6
During any visit to a doctor or other health worker in the past 12 months, were you advised to quit smoking tobacco?	Yes<br>No<br>No visit during the past 12 months	1 *If T2=Yes, go to T12; if T2=No, goto T9*<br>2 *If T2=Yes, go to T12; if T2=No, go to T9*<br>3 *If T2=Yes, go to T12; if T2=No, go to T9*	T7
In the past, did you **eversmoke** any tobacco products? *(USE SHOWCARD)*	Yes<br>No	1<br>2 *If No, go to T12*	T8
In the past, did you **ever** smoke **daily**?	Yes<br>No	1 *If T1=Yes, go to T12, else go to T10*<br>2*If T1=Yes, go to T12, else go to T10*	T9

How old were you when you **stopped** smoking?	Age (years)<br>Don't Know 77	└─┴─┘ *If Known, go to T12*	T10
How **long ago** did you stop smoking?<br>*(RECORD ONLY 1, NOT ALL 3)*<br>*Don't Know 77*	Years ago	└─┴─┘ *If Known, go to T12*	T11a
	OR Months ago	└─┴─┘ *If Known, go to T12*	T11b
	OR Weeks ago	└─┴─┘	T11c
Do you **currently use** any **smokeless tobacco** products such as *[snuff, chewing tobacco, tuibu, gutka, betel, Pan masala]*? *(USE SHOWCARD)*	Yes	1	T12
	No	2 *If No, go to T15*	
Do you **currently usesmokeless tobacco** products **daily?**	Yes	1	T13
	No	2 *If No, go to T14aw*	
On average, how many **times a day/week** do you use ….<br>*(IF LESS THAN DAILY, RECORD WEEKLY)*<br>*(RECORD FOR EACH TYPE, USE SHOWCARD)*<br>*Don't Know 7777*		DAILY↓ WEEKLY↓	
	Tobacco Snuff, by mouth	└─┴─┴─┴─┘ └─┴─┴─┴─┘	T14a/ T14aw
	Snuff, by nose	└─┴─┴─┴─┘ └─┴─┴─┴─┘	T14b/ T14bw
	Chewing tobacco	└─┴─┴─┴─┘ └─┴─┴─┴─┘	T14c/ T14cw
	Betel, quid with tobacco	└─┴─┴─┴─┘ └─┴─┴─┴─┘	T14d/ T14dw
	Other	└─┴─┴─┴─┘ └─┴─┴─┴─┘ *If Other, go to T14other, if T13=No, go to T16, else go to T17*	T14e/ T14ew
	Other (please specify):	└─┴─┴─┴─┴─┴─┘ *If T13=No, go to T16, else go to T17*	T14other/ T14otherw

In the **past**, did you **ever use** smokeless tobacco products such as *[snuff, chewing tobacco, gutka or betel with tobacco, pan masala]*?	Yes	1	T15
	No	2 *If No, go to T17*	
In the **past**, did you **ever use** smokeless tobacco products such as *[snuff, chewing tobacco, gutka,betel with tobacco* **daily**?	Yes	1	T16
	No	2	
During the past 30 days, did someone smoke **in your home**?	Yes	1	T17
	No	2	
During the past 30 days, did someone smoke in closed areas **in your workplace** (in the building, in a work area or a specific office)?	Yes	1	T18
	No	2	
	Don't work in a closed area	3	

Alcohol Consumption

The next questions ask about the consumption of alcohol.

Question	Response		Code
Have you **ever** consumed any alcohol such as beer, wine, whisky spirits or *locally prepared alcohol*? *(USE SHOWCARD OR SHOW EXAMPLES)*	Yes	1	A1
	No	2 *If No, go to A16*	
Have you consumed any alcohol within the **past 12 months**?	Yes	1 *If Yes, go to A4*	A2
	No	2	
Have you stopped drinking due to health reasons, such as a negative impact on your health or on the advice of your doctor or other health worker?	Yes	1 *If Yes, go to A16*	A3
	No	2 *If No, go to A16*	
During the past 12 months, **how frequently** have you had at least one standard alcoholic drink? *(READ RESPONSES, USE SHOWCARD)*	Daily	1	A4
	5-6 days per week	2	
	3-4 days per week	3	
	1-2 days per week	4	
	1-3 days per month	5	
	Less than once a month	6	
Have you consumed any alcohol within the **past 30 days**?	Yes	1	A5
	No	2 *If No, go to A13*	
During the past 30 days, on how many **occasions** did you have at least one standard alcoholic drink?	Number<br>Don't know 77	└─┴─┘	A6
During the past 30 days, when you drank alcohol, how many **standarddrinks on average** did you have during one drinking occasion? *(USE SHOWCARD)*	Number<br>Don't know 77	└─┴─┘	A7
During the past 30 days, what was the **largest number** of standard drinks you had on a single occasion, counting all types of alcoholic drinks together?	Largest number<br>Don't Know 77	└─┴─┘	A8
During the past 30 days, how many times did you have **six or more** standard drinks in a single drinking occasion?	Number of times<br>Don't Know 77	└─┴─┘	A9
During each of the **past 7 days**, how many standard drinks did you have each day? *(USE SHOWCARD)* *Don't Know 77*	Monday	└─┴─┘	A10a
	Tuesday	└─┴─┘	A10b
	Wednesday	└─┴─┘	A10c
	Thursday	└─┴─┘	A10d
	Friday	└─┴─┘	A10e
	Saturday	└─┴─┘	A10f
	Sunday	└─┴─┘	A10g

I have just asked you about your consumption of alcohol during the past 7 days. The questions were about alcohol in general, while the next questions refer to your consumption of homebrewed alcohol, alcohol brought over the border/from another country, any alcohol not intended for drinking or other untaxed alcohol. Please only think about these types of alcohol when answering the next questions.			
**Question**	**Response**		**Code**
During the **past 7 days**, did you consume any **homebrewed** alcohol, any alcohol **brought over the border/from another country**, any alcohol **not intended for drinking** or other **untaxed** alcohol? *(USE SHOWCARD)*	Yes	1	A11
	No	2 *If No, go to A13*	
On average, **how many standard drinks** of the following did you consume **during the past 7 days**? *(USE SHOWCARD)* *Don't Know 77*	DesiSharab (santra, khatta, Malwa) or Homebrewed spirits	└─┴─┘	A12a
	Homebrewed beer or wine, e.g. beer, or fruit wine	└─┴─┘	A12b
	Alcohol brought over the border/from another country	└─┴─┘	A12c
	Alcohol not intended for drinking, e.g. alcohol-based medicines, perfumes, after shaves	└─┴─┘	A12d
	Other untaxed alcohol in the country	└─┴─┘	A12e
During the **past 12 months,** how often have you found that you were not able to stop drinking once you had started?	Daily or almost daily	1	A13
	Weekly	2	
	Monthly	3	
	Less than monthly	4	
	Never	5	
During the **past 12 months,** how often have you failed to do what was normally expected from you because of drinking?	Daily or almost daily	1	A14
	Weekly	2	
	Monthly	3	
	Less than monthly	4	
	Never	5	
During the **past 12 months,** how often have you needed a first drink in the morning to get yourself going after a heavy drinking session?	Daily or almost daily	1	A15
	Weekly	2	
	Monthly	3	
	Less than monthly	4	
	Never	5	
During the **past 12 months**, have you had family problems or problems with your partner due to **someone else's** drinking?	Yes, more than monthly	1	A16
	Yes, monthly	2	
	Yes, several times but less than monthly	3	
	Yes, once or twice	4	
	No	5	

Diet		
The next questions ask about the fruits and vegetables that you usually eat. I have a nutrition card here that shows you some examples of local fruits and vegetables. Each picture represents the size of a serving. As you answer these questions please think of a typical week in the last year.		
**Question**	**Response**	**Code**
In a typical week, on how many days do you **eat fruit**? *(USE SHOWCARD)*	Number of days<br>Don't Know 77 └─┴─┘ *If Zero days, go to D3*	D1
How many **servings** of fruit do you eat on **one** of those days? *(USE SHOWCARD)*	Number of servings<br>Don't Know 77 └─┴─┘	D2
In a typical week, on how many days do you **eat vegetables**? *(USE SHOWCARD)*	Number of days<br>Don't Know 77 └─┴─┘ *If Zero days, go to D5*	D3
How many **servings** of vegetables do you eat on one of those days? *(USE SHOWCARD)*	Number of servings<br>Don't know 77 └─┴─┘	D4

Dietary Salt		
With the next questions, we would like to learn more about salt in your diet. Dietary salt includes ordinary table salt, unrefined salt such as sea salt, iodized salt, and powders, and salty sauces such as soya sauce or fish sauce (see showcard). The following questions are on adding salt to the food right before you eat it, on how food is prepared in your home, on eating processed foods that are high in salt such as *pickles, chutneys*, and questions on controlling your salt intake. Please answer the questions even if you consider yourself to eat a diet low in salt.		
**Question**	**Response**	**Code**
How often do you **add salt or a salty sauce such as soya sauce** to your food right before you eat it or as you are eating it?<br>*(SELECT ONLY ONE)*<br>*(USE SHOWCARD)*	Always 1<br>Often 2<br>Sometimes 3<br>Rarely 4<br>Never 5<br>Don't know 77	D5
How often is **salt, salty seasoning or a salty sauce added** in cooking or preparing foods in your household?	Always 1<br>Often 2<br>Sometimes 3<br>Rarely 4<br>Never 5<br>Don't know 77	D6
How often do you eat **processed food high in salt**? By processed food high in salt, I mean foods that have been altered from their natural state, such as packaged salty snacks, canned salty food, salty food prepared at a fast food restaurant, pickles, chutneys	Always 1<br>Often 2<br>Sometimes 3<br>Rarely 4<br>Never 5<br>Don't know 77	D7
**How much salt or salty sauce** do you think you consume?	Far too much 1<br>Too much 2<br>Just the right amount 3<br>Too little 4	D8

	Far too little	5	
	Don't know	77	
How important to you is **lowering the salt** in your diet?	Very important	1	D9
	Somewhat important	2	
	Not at all important	3	
	Don't know	77	
Do you think that too much salt or salty sauce in your diet could cause a **health problem**?	Yes	1	D10
	No	2	
	Don't know	77	
Do you do any of the following on a regular basis to **control your salt intake**? *(RECORD FOR EACH)*			
Limit consumption of processed foods	Yes	1	D11a
	No	2	
Look at the salt or sodium content on food labels	Yes	1	D11b
	No	2	
Buy low salt/sodium alternatives	Yes	1	D11c
	No	2	
Use spices other than salt when cooking	Yes	1	D11d
	No	2	
Avoid eating foods prepared outside of a home	Yes	1	D11e
	No	2	
Do other things specifically to control your salt intake	Yes	1 *If Yes, go to D11other*	D11f
	No	2	
Other (please specify)	|__|__|__|__|__|__|__|		D11other

What type of **oil or fat is most often** used for meal preparation in your household? *(USE SHOWCARD)* *(SELECT ONLY ONE)*	Mustard oil	1	D12
	Soyabean oil	2	
	Butter or Pure ghee	3	
	Sunflower oil	4	
	Other	5 *If Other, go to D12 other*	
	None in particular	6	
	None used	7	
	Don't know	77	
	Other	|__|__|__|__|__|__|__|	D12other
On average, how many meals per week do you eat that were not prepared at a home? By meal, I mean breakfast, lunch and dinner.	Number<br>Don't know 77	|__|__|	D13

Physical Activity		
Next I am going to ask you about the time you spend doing different types of physical activity in a typical week. Please answer these questions even if you do not consider yourself to be a physically active person. Think first about the time you spend doing work. Think of work as the things that you have to do such as paid or unpaid work, study/training, household chores, harvesting food/crops, fishing or hunting for food, seeking employment.In answering the following questions 'vigorous-intensity activities' are activities that require hard physical effort and cause large increases in breathing or heart rate, 'moderate-intensity activities' are activities that require moderate physical effort and cause small increases in breathing or heart rate.		
**Question**	**Response**	**Code**
**Work**		
Does your work involve vigorous-intensity activity that causes large increases in breathing or heart rate like *[carrying or liftingheavy loads, digging or construction work]* for at least 10 minutes continuously? *(USE SHOWCARD)*	Yes 1<br>No 2 *If No, go to P 4*	P1
In a typical week, on how many days do you do vigorous-intensity activities as part of your work?	Number of days └─┘	P2
How much time do you spend doing vigorous-intensity activities at work on a typical day?	Hours : minutes └─┴─┘ : └─┴─┘ hrsmins	P3 (a-b)
Does your work involve moderate-intensity activity, that causes small increases in breathing or heart rate such as brisk walking, *carrying light loads, manual washing of clothes, dry sweeping of floor, wet mopping of floor, drawing water from well carrying water from tap, carrying water from river or well, manual grinding or pounding of cereals, gardening at home, carrying groceries from market* for at least 10 minutes continuously? *(USE SHOWCARD)*	Yes 1<br>No 2 *If No, go to P 7*	P4
In a typical week, on how many days do you do moderate-intensity activities as part of your work?	Number of days └─┘	P5
How much time do you spend doing moderate-intensity activities at work on a typical day?	Hours : minutes └─┴─┘ : └─┴─┘ hrsmins	P6 (a-b)
**Travel to and from places**		
The next questions exclude the physical activities at work that you have already mentioned. Now I would like to ask you about the usual way you travel to and from places. For example to work, for shopping, to market, to place of worship.		
Do you walk or use a bicycle *(pedal cycle)* for at least 10 minutes continuously to get to and from places?	Yes 1<br>No 2 *If No, go to P 10*	P7
In a typical week, on how many days do you walk or bicycle for at least 10 minutes continuously to get to and from places?	Number of days └─┘	P8
How much time do you spend walking or bicycling for travel on a typical day?	Hours : minutes └─┴─┘ : └─┴─┘ hrsmins	P9 (a-b)

Recreational activities		
The next questions exclude the work and transport activities that you have already mentioned.<br>Now I would like to ask you about sports, fitness and recreational activities (leisure)		
Do you do any vigorous-intensity sports, fitness or recreational *(leisure)* activities that cause large increases in breathing or heart rate like *[running or football]* for at least 10 minutes continuously?<br>*(USE SHOWCARD)*	Yes 1<br>No 2 *If No, go to P 13*	P10
In a typical week, on how many days do you do vigorous-intensity sports, fitness or recreational *(leisure)* activities?	Number of days └─┘	P11
How much time do you spend doing vigorous-intensity sports, fitness or recreational activities on a typical day?	Hours : minutes └─┴─┘ : └─┴─┘<br>hrsmins	P12 (a-b)
Do you do any moderate-intensity sports, fitness or recreational *(leisure)* activities that cause a small increase in breathing or heart rate such as brisk walking, *[cycling, swimming, volleyball]* for at least 10 minutes continuously?<br>*(USE SHOWCARD)*	Yes 1<br>No 2 *If No, go to P16*	P13
In a typical week, on how many days do you do moderate-intensity sports, fitness or recreational *(leisure)* activities?	Number of days └─┘	P14
How much time do you spend doing moderate-intensity sports, fitness or recreational *(leisure)* activities on a typical day?	Hours : minutes └─┴─┘ : └─┴─┘<br>hrsmins	P15 (a-b)

Sedentary behaviour			
The following question is about sitting or reclining at work, at home, getting to and from places, or with friends including time spent sitting at a desk, sitting with friends, traveling in car, bus, train, reading, playing cards or watching television, but do not include time spent sleeping. *(USE SHOWCARD)*			
How much time do you usually spend sitting or reclining on a typical day?	Hours : minutes	└─┴─┘ : └─┴─┘<br>hrsmins	P16 (a-b)

History of Raised Blood Pressure		
**Question**	**Response**	**Code**
Have you ever had your blood pressure measured by a doctor or other health worker?	Yes 1<br>No 2 *If No, go to H6*	H1
Have you ever been told by a doctor or other health worker that you have raised blood pressure or hypertension?	Yes 1<br>No 2 *If No, go to H6*	H2a
Have you been told in the past 12 months?	Yes 1<br>No 2	H2b
In the past two weeks, have you taken any drugs (medication) for raised blood pressure prescribed by a doctor or other health worker?	Yes 1<br>No 2	H3

Have you ever seen a traditional healer for raised blood pressure or hypertension?	Yes 1<br>No 2	H4
Are you currently taking any herbal or traditional remedy for your raised blood pressure?	Yes 1<br>No 2	H5

History of Diabetes		
Have you ever had your blood sugar measured by a doctor or other health worker?	Yes 1<br>No 2 *If No, go to H12*	H6
Have you ever been told by a doctor or other health worker that you have raised blood sugar or diabetes?	Yes 1<br>No 2 *If No, go to H12*	H7a
Have you been told in the past 12 months?	Yes 1<br>No 2	H7b
In the past two weeks, have you taken any drugs (medication) for diabetes prescribed by a doctor or other health worker?	Yes 1<br>No 2	H8
Are you currently taking insulin for diabetes prescribed by a doctor or other health worker?	Yes 1<br>No 2	H9
Have you ever seen a traditional healer for diabetes or raised blood sugar?	Yes 1<br>No 2	H10
Are you currently taking any herbal or traditional remedy for your diabetes?	Yes 1<br>No 2	H11

History of Raised Total Cholesterol

Question	Response	Code
Have you ever had your cholesterol (fat levels in your blood) measured by a doctor or other health worker?	Yes 1<br>No 2 *If No, go to H17*	H12
Have you ever been told by a doctor or other health worker that you have raised cholesterol?	Yes 1<br>No 2 *If No, go to H17*	H13a
Have you been told in the past 12 months?	Yes 1<br>No 2	H13b
In the past two weeks, have you taken any oral treatment (medication) for raised total cholesterol prescribed by a doctor or other health worker?	Yes 1<br>No 2	H14
Have you ever seen a traditional healer for raised cholesterol?	Yes 1<br>No 2	H15
Are you currently taking any herbal or traditional remedy for your raised cholesterol?	Yes 1<br>No 2	H16

History of Cardiovascular Diseases			
Have you ever had a heart attack or chest pain from heart disease (angina) or a stroke (cerebrovascular accident or incident)?	Yes<br>No	1<br>2	H17
Are you currently taking aspirin regularly to prevent or treat heart disease?	Yes<br>No	1<br>2	H18
Are you currently taking statins (Lovastatin/Simvastatin/Atorvastatin or any other statin) regularly to prevent or treat heart disease?	Yes<br>No	1<br>2	H19

History of Chronic Kidney Diseases			
Have you ever been told by a doctor or other health care provider that you have kidney stones?	Yes<br>No	1<br>2	X1
Have you ever been told by a doctor or other health care provider that you have kidney disease, weak kidneys or low kidney function?	Yes<br>No	1<br>2	X2
Have you ever been on dialysis or had a kidney transplant?	Yes<br>No	1<br>2	X3

Lifestyle Advice			
During the past three years, has a doctor or other health worker advised you to do any of the following? *(RECORD FOR EACH)*			
Quit using tobacco or don't start	Yes<br>No	1<br>2	H20a
Reduce salt in your diet	Yes<br>No	1<br>2	H20b
Eat at least five servings of fruit and/or vegetables each day	Yes<br>No	1<br>2	H20c
Reduce fat in your diet	Yes<br>No	1<br>2	H20d
Start or do more physical activity	Yes<br>No	1<br>2	H20e
Maintain a healthy body weight or lose weight	Yes<br>No	1 *If C1=2 go to CX1*<br>2	H20f

Health screening		
**Cervical cancer**		
The next question asks about cervical cancer prevention. Screening tests for cervical cancer prevention can be done in different ways, including Visual Inspection with Acetic Acid/vinegar (VIA), pap smear and Human Papillomavirus (HPV) test. VIA is an inspection of the surface of the uterine cervix after acetic acid (or vinegar) has been applied to it. For both pap smear and HPV test, a doctor or nurse uses a swab to wipe from inside your vagina, take a sample and send it to a laboratory. It is even possible that you were given the swab yourself and asked to swab the inside of your vagina. The laboratory checks for abnormal cell changes if a pap smear is done, and for the HP virus if an HPV test is done.		
**Question**	**Response**	**Code**
**<u>The following questions are for women only:</u>**<br><br>Have you ever had a screening test for cervical cancer, using any of these methods described above?	Yes 1<br>No 2<br>Don't know 77	CX1

BREAST CANCER			
**<u>The following questions (S1-S3) are for women only:</u>**<br>Have you been shown or taught how to examine your breasts?	Yes<br>No	1<br>2	X4
When was the last time you had an examination of your breasts?	1 year or less<br>Between 1 and 2 years<br>More than 2 years<br>Never<br>Don't know	1<br>2<br>3<br>4<br>77	X5
When was the last time you had a mammogram?	1 year or less<br>Between 1 and 2 years<br>More than 2 years<br>Never<br>Don't know	1<br>2<br>3<br>4<br>77	X6
**ORAL CANCER**			
Have you ever had lining/covering of your mouth and tongue examined by a dentist or a doctor?"	Yes	1	X7
	No	2	
	Don't know	77	
	Refused	88	
When did you have your most recent oral or mouth cancer exam	1 year or less	1	X8
	Between 1 and 2 years	2	
	More than 2 years	3	
	Never	4	
	Don't know	77	

Family history			
**Question**	**Response**		**Code**
Have some of your family members been diagnosed with the following diseases?			
Diabetes or raised blood sugar	Yes<br>No	1<br>2	F1A
Raised Blood pressure	Yes<br>No	1<br>2	F1B
Stroke	Yes<br>No	1<br>2	F1C
Cancer or malignant tumor	Yes<br>No	1<br>2	F1D
Raised Cholesterol	Yes<br>No	1<br>2	F1E
Early Heart attack (below age 55 for men and below age 65 for women)	Yes<br>No	1<br>2	F1f
Chronic Kidney Diseases	Yes<br>No	1<br>2	F1g

`Mental health / Suicide		
**Over the last 2 weeks, how often have you been bothered by any of the following problems?**<br>**The next questions ask about thoughts, plans, and attempts of suicide. Please answer the questions even if no one usually talks about these issues.**		
**Question**	**Response**	**Code**
During the **past 2 weeks**, have you had little interest or pleasure in doing things	Not at all 0<br>Several days 1<br>More than half days 2<br>Nearly every day 3	MHX1
During the **past 2 weeks**, have you felt down, depressed, or hopeless	Not at all 0<br>Several days 1<br>More than half days 2<br>Nearly every day 3	MHX2
During the **past 2 weeks**, have you had trouble falling or staying asleep, or sleeping too much	Not at all 0<br>Several days 1<br>More than half days 2<br>Nearly every day 3	MHX3
During the **past 2 weeks**, are you feeling tired or having little energy	Not at all 0<br>Several days 1<br>More than half days 2<br>Nearly every day 3	MHX4
During the **past 2 weeks**, are you overeating or have a poor appetite	Not at all 0<br>Several days 1<br>More than half days 2<br>Nearly every day 3	MHX5
During the **past 2 weeks**, are you feeling bad about yourself — or that you are a failure or have let yourself or your family down	Not at all 0<br>Several days 1<br>More than half days 2<br>Nearly every day 3	MHX6
During the **past 2 weeks**, are you having trouble concentrating on things, such as reading the newspaper or watching television	Not at all 0<br>Several days 1<br>More than half days 2<br>Nearly every day 3	MHX7
During the **past 2 weeks**, are you moving or speaking so slowly that other people could have noticed? Or the opposite — being so fidgety or restless that you have been moving around a lot more than usual	Not at all 0<br>Several days 1<br>More than half days 2<br>Nearly every day 3	MHX8
During the **past 2 weeks**, have you had thoughts that you would be better off dead or of hurting yourself in some way	Not at all 0<br>Several days 1<br>More than half days 2<br>Nearly every day 3	MHX9
During the **past 12 months**, have you seriously **considered** attempting suicide?	Yes 1<br>No 2*If No, go to MH3*<br>Refused 88	MH1
Did you seek **professional help** for these thoughts?	Yes 1<br>No 2<br>Refused 88	MH2
During the **past 12 months**, have you made **a plan about how** you would attempt suicide?	Yes 1<br>No 2	MH3

	Refused	88	
Have you **ever attempted suicide**?	Yes<br>No<br>Refused	1<br>2*If No, go to MH9*<br>88	MH4
During the **past 12 months**, have you **attempted suicide**?	Yes<br>No<br>Refused	1<br>2<br>88	MH5
What was the main **method you used** the last time you attempted suicide?<br><br>*(SELECT ONLY ONE)*	Razor, knife or other sharp instrument<br>Overdose of medication (e. g. prescribed, over-the-counter)<br>Overdose of other substance (e.g. heroin, crack, alcohol)<br>Poisoning with pesticides (e.g. rat poison, insecticide, weed-killer)<br>Other poisoning (e.g. plant/seed, household product)<br>Poisonous gases from charcoal<br>Other<br>Refused	1<br>2<br>3<br>4<br>5<br>6<br>7 *If Other, go to MH6other*<br>88	MH6
	Other (specify)	└─┴─┴─┴─┴─┴─┴─┘	MH6other
Did you seek **medical care** for this attempt?	Yes<br>No<br>Refused	1<br>2 *If No, go to MH9*<br>88	MH7
Were you **admitted to hospital overnight** because of this attempt?	Yes<br>No<br>Refused	1<br>2<br>88	MH8
Has anyone in **your close family** (mother, father, brother, sister or children) ever attempted suicide?	Yes<br>No<br>Refused	1<br>2<br>88	MH9
Has anyone in **your close family** (mother, father, brother, sister or children) ever died from suicide?	Yes<br>No<br>Refused	1<br>2<br>88	MH10

Health Care

Next I am going to ask you about your health insurance coverage and your use of health services in relation to any noncommunicable disease (NCD) you may have. NCDs include cardiovascular diseases (such as heart diseases, cerebrovascular disease and stroke, peripheral arterial disease, and deep vein thrombosis and pulmonary embolism), cancers, chronic respiratory diseases (such as asthma, chronic obstructive pulmonary disease, occupational lung diseases or pulmonary hypertension) and diabetes.

Health Care Coverage

Please provide information about your current health insurance coverage. Health insurance coverage means being enrolled with an organization that pays for health care costs if you get sick or injured.

Do you currently have **health insurance**?	Yes<br>No	1<br>2 *If No, go to HC3*	HC1
**What kind** of **health insurance** do you currently have?	Mandatory health insurance plan<br>Voluntary health insurance plan<br>Other	1<br>2<br>3*If Other, go to HC2other*	HC2

(CGHS, RSBY, ESI, PRIVATE HEALTH INSURANCE)	Don't know	77	
	Other (please specify):	└┴┴┴┴┴┴┘	HC2 other
During the **past 12 months**, which of the following **financial sources** did you use to pay for any **health expenditures** such as medicines, consultations, treatment, hospitalization or patient care?	Current income of any household members	Yes 1<br>No 2	HC3a
	Savings (e. g. bank account)	Yes 1<br>No 2	HC3b
	Payment or reimbursement from a health insurance plan	Yes 1<br>No 2	HC3c
	Sold items (e. g. furniture, animals, jewellery)	Yes 1<br>No 2	HC3d
	Family members or friends from outside the household	Yes 1<br>No 2	HC3e
	Borrowed from someone other than a friend or family	Yes 1<br>No 2	HC3f
	Other	Yes 1 *If Other, go to [illegible]*<br>No 2	HC3g
	Other (please specify):	└┴┴┴┴┴┴┘	HC3 other

Health Care Utilization

Please think about your visits to any health center and the treatments you received there which were related to an NCD you may have.

Have you ever had or do you currently have **a non-communicable disease (NCD)** such as cardiovascular disease including heart disease and stroke, cancer, chronic respiratory disease, or diabetes?	Yes<br>No	1<br>2 *If No, go to [next section]*	HC4
	If yes, Please specify the name of NCD		HC4X
Have you ever visited any **health care facility**due to anNCDyou have? Please exclude any hospitalization.	Yes<br>No	1<br>2 *If No, go to HC11*	HC5
How much **time** did you spend **traveling** the **last time** you visited a health care facility (taking both ways into account)?<br>*Don't know 77:77:77*	Days : hours : minutes	└┴┘ : └┴┘ : └┴┘<br>days hrsmins	HC6
How long was the **waiting time** before your appointment started when you **last** visited a health care facility?<br>*Don't know 77:77*	Hours : minutes	└┴┘ : └┴┘<br>hrsmins	HC7
During the **past 30 days**, have youvisitedany health care facility due to anNCD you have? Please exclude any hospitalization.	Yes<br>No	1<br>2 *If No, go to HC11*	HC8

During the **past 30 days**, how many times have you visited a health care facility due to an NCD you have?<br>*(RECORD FOR EACH)*<br>*[INSERT COUNTRY-SPECIFIC CATEGORIES]*<br>*Don't know 77*	Health Center	└┴┘	HC9a
	Public Hospital	└┴┘	HC9b
	Private Hospital	└┴┘	HC9c
	Doctor's Office	└┴┘	HC9d
	Other	└┴┘ *If Other, go to HC9other*	HC9e

	Other (please specify):	└─┴─┴─┴─┴─┴─┘	HC9otehr
During the **past 30 days**, taking all your visits to a health care facility due to an NCD into account, **how much did you pay yourself** for these visits in total?<br><br>*(RECORD FOR EACH OR PUT TOTAL AMOUNT)*<br>*Don't know 77777*	Health care provider's fees	└─┴─┴─┴─┴─┘ [INR]	HC10a
	Medicines	└─┴─┴─┴─┴─┘ [INR]	HC10b
	Tests	└─┴─┴─┴─┴─┘ [INR]	HC10c
	Transport	└─┴─┴─┴─┴─┘ [INR]	HC10d
	Other	└─┴─┴─┴─┴─┘ [INR]	HC10e
	**OR Total Amount**	└─┴─┴─┴─┴─┘ [INR]	HC10f
**During the past 30 days, how much did you pay yourself** for health care **not** related to any visit of a health care facility or hospital, such as routine medication?<br>*Don't know 77777*	**Amount**	└─┴─┴─┴─┴─┘ [INR]	HC11
During the **past 12 months**, have you been **hospitalized** due to an NCD?	Yes<br>No	1<br>2 *If No, go to HC15*	HC12
During the **past 12 months**, **how many days** have you been hospitalized due to an NCD?<br>*Don't know 777*	Number of days	└─┴─┴─┘	HC13
During the **past 12 months**, taking all your visits to a hospital due to an NCD into account, **how much did you pay yourself** for these visits in total?<br><br>*(RECORD FOR EACH OR PUT TOTAL AMOUNT)*<br>*Don't know 77777*	Health care provider's fees	└─┴─┴─┴─┴─┘ [INR]	HC14a
	Medicines	└─┴─┴─┴─┴─┘ [INR]	HC14b
	Tests	└─┴─┴─┴─┴─┘ [INR]	HC14c
	Transport	└─┴─┴─┴─┴─┘ [INR]	HC14d
	Other	└─┴─┴─┴─┴─┘ [INR]	HC14e
	**OR Total Amount**	└─┴─┴─┴─┴─┘ [INR]	HC14f
**Home Care**			
Please think about home care from family membersand/or friends because of an NCD you have.			
During the **past 30 days**, has a **family member or friend provided care** for you at home due to your NCD?	Yes<br>No	1<br>2 *If No, go to HC17*	HC15
During the **past 30 days**, how many **hours per week** has this person/have these people provided care for you?<br>*Don't know 777*	Hours per week	└─┴─┴─┘<br>hrs	HC16
**Loss of Productivity**			
Please think about the time you couldn't do your usual activity (for example, work, work at home, study) because of an NCD you have.			
During the **past 30 days**, have you **missed any time of your usual activity** (work, work at home, study) due to an NCD?	Yes<br>No	1<br>2 *If No, go to [next section]*	HC17
During the **past 30 days**, **how many days** of your usual activity have you missed due to an NCD?<br>*Don't know 77*	Number of days	└─┴─┘<br>days	HC18

Physical Measurements

Blood Pressure

Question		Response	Code
Interviewer ID		└─┴─┴─┘	M1
Device ID for blood pressure		└─┴─┘	M2
Reading 1	Systolic (mmHg)	└─┴─┴─┘	M4a
	Diastolic (mmHg)	└─┴─┴─┘	M4b
Reading 2	Systolic (mmHg)	└─┴─┴─┘	M5a
	Diastolic (mmHg)	└─┴─┴─┘	M5b
Reading 3	Systolic (mmHg)	└─┴─┴─┘	M6a
	Diastolic (mmHg)	└─┴─┴─┘	M6b
During the past two weeks, have you been treated for raised blood pressure with drugs (medication) prescribed by a doctor or other health worker?	Yes<br>No	1<br>2	M7

Height and Weight

Question		Response	Code
Interviewer ID		└─┴─┴─┘	M9
Device IDs for height and weight	Height<br>Weight	└─┴─┘<br>└─┴─┘	M10a<br>M10b
Height	in Centimetres (cm)	└─┴─┴─┘.└─┘	M11
Weight<br>*If too large for scale 666.6*	in Kilograms (kg)	└─┴─┴─┘.└─┘	M12

Waist

Question		Response	Code
Device ID for waist		└─┴─┘	M13
Waist circumference	in Centimetres (cm)	└─┴─┴─┘.└─┘	M14

Hip Circumference and Heart Rate

Question		Response	Code
Hip circumference	in Centimeters (cm)	└─┴─┴─┘.└─┘	M15
Heart Rate			
Reading 1	Beats per minute	└─┴─┴─┘	M16a
Reading 2	Beats per minute	└─┴─┴─┘	M16b

Reading 3	Beats per minute └─┴─┴─┘	M16c
**Skin Fold Thickness**		
Triceps	in millimetres (mm) └─┴─┴─┘.└─┘	MR2X
Suprailiac	in millimetres (mm) └─┴─┴─┘.└─┘	MR3X
Subscapular	in millimetres (mm) └─┴─┴─┘.└─┘	MR4X

Biochemical Measurements

Blood Glucose

Question	Response	Code
During the past 12 hours have you had anything to eat or drink, other than water?	Yes 1<br>No 2	B1
Technician/interviewer ID	└─┴─┴─┘	B2
Device ID	└─┴─┘	B3
Time of day blood specimen taken (24 hour clock)	Hours : minutes └─┴─┘:└─┴─┘<br>hrsmins	B4
Fasting blood glucose	mg/dl └─┴─┘.└─┴─┘	B5
Today, have you taken insulin or other drugs (medication) that have been prescribed by a doctor or other health worker for raised blood glucose?	Yes 1<br>No 2	B6
**Blood Sample for total cholesterol, triglycerides and serum creatinine**		
Sample ID	└─┴─┘	B7
Total cholesterol	mg/dl └─┴─┘.└─┴─┘	B8
During the past two weeks, have you been treated for raised cholesterol with drugs (medication) prescribed by a doctor or other health worker?	Yes 1<br>No 2	B9
Triglycerides	mg/dl └─┴─┘.└─┴─┘	B16
Serum creatinine	mg/dl └─┴─┴─┘.└─┘	*BX 17*

Urinary sodium, albumin and creatinine

Had you been fasting prior to the urine collection?	Yes 1	B10

	No	2	
Technician ID		└─┴─┴─┘	B11
Sample ID		└─┴─┘	B12
Time of day urine sample taken (24 hour clock)	Hours : minutes	└─┴─┘ : └─┴─┘<br>hrsmins	B13
Urinary sodium	mg/dl	└─┴─┴─┘ . └─┘	B14
Urinary creatinine	mg/dl	└─┴─┘ . └─┴─┘	B15
Urinary albumin	mg/dl	└─┴─┴─┘ . └─┘	BX16
Albumin creatinine ratio	Mg/g		BX18
Serum Cystatin C	Mg/l		BX 19
Serum High Density Cholesterol	mg/dl		BX 20
Serum Low Density Cholesterol	mg/dl		BX 21
