# Supplementary material for: Non-communicable diseases risk factors and their determinants: A cross-sectional state-wide STEPS survey, Haryana, North India
Source: PLoS One. 2019 Nov 27;14(11):e0208872. doi: 10.1371/journal.pone.0208872 (PMC6881003; doi:10.1371/journal.pone.0208872)
Supplement: S3 File — (PDF) [file pone.0208872.s004.pdf]

Annexure 1.5: Study instrument

हरियाणा में गैर-संचारी बीमारियों के कारणों का सर्वेक्षण

सर्वेक्षण जानकारी		
स्थान और तिथी	जवाब	Code
यूनिक आई. डी	__ ___ __ ___ ___	I1
गाँव/सी. ई. बी का नाम		I2
मुलाकात करने वाले की आई. डी	└─┴─┘	I3
फॉर्म पूरा करने की तिथी	└─┴─┘ └─┴─┘ └─┴─┴─┴─┘<br>दिन महीना साल	I4

सहमति, मुलाकात की भाषा और नाम	जवाब	Code
सहमति पत्र पढ़कर प्राप्त किया हुआ	हाँ। 1<br>ना 2 यदि ना तो अंत	I5
मुलाकात की भाषा	अंग्रेजी 1<br>हिंदी 2<br>पंजाबी/ 3<br>अन्य 4	I6
मुलाकात का समय<br>(24 hour clock)	└─┴─┘ : └─┴─┘<br>hrsmins	I7
परिवार का उपनाम		I8
पहला नाम		I9
फोन नं		I10
12 अंको का आधार आई. डी नम्बर		I11

जन-आँकड़ा जानकारी		
प्रश्न	जवाब	Code
लिंग (पुरूष-औरत)	पुरूष 1<br>[illegible] 2	C1

जन्म तिथि<br>पता नहीं 77 77 7777	⎣__⎦ ⎣__⎦ ⎣__⎦<br>दिन महीना साल	C2
आपकी उम्र कितनी है।	महीना ⎣__⎦	C3
आपने कितने वर्ष स्कूल और पूरा समय अध्ययन में व्यतीत किया है।	साल ⎣__⎦	C4

आपने किस उच्चतम कक्षा तक शिक्षा प्राप्त की हैं।	गैर स्कूल शिक्षा	1	C5
	प्राइमरी स्कूल से कम	2	
	आपका समाजिक समूह क्या है।	3	
	सैकेड़री स्कूल तक	4	
	हाई स्कूल तक	5	
	कॉलेज यूनिवर्सिटी तक	6	
	पोस्ट ग्रेजुएट डिग्री	7	
	जवाब देने से इंकार /कुछ नहीं	88	
आपका समाजिक समूह क्या है।	पिछड़ी जाति	1	C6
	दूसरी पिछड़ी जाति	2	
	सामान्य	3	
	कुछ नहीं	88	
आपका जातीय समूह क्या है।	जाट		R1E
	यदुवंशी		
	अहिर		
	कम्बोजी		
	गुज्जर		
	ब्राह्मण		
	राजपूत		
	शेर (रोड)		
	सैनी		
	पंजाबी		
	अन्य..................		
आपकी वैवाहिक स्थिति क्या हैं।	अविवाहित	**1**	C7
	विवाहित	2	
	अलग	3	
	तलाकशुदा	4	
	विधवा	5	
	साथ में रह रहे हैं	6	
	जवाब देन से इनकार	88	
निम्नलिखित कामों में से कौन सा काम पिछले आपके 12 महीनों में आपके काम की स्थिति को दर्शाते हैं।	सरकारी कर्मचारी	1	C8
	गैर-सरकारी कर्मचारी	2	
	स्व: रोजगार	3	
	बिना पैसों के काम	4	
	विद्यार्थी	5	
	घरेलू काम करने वाले	6	
	सेवा मुकत	7	
	बेरोजगार	8	
	काम न करने योग्य	9	
	जवाब देने से इंकार	88	

आपके घर में आपको मिलाके कितने व्यक्ति 18 साल से ऊपर है।	लागों की गिनती	└─┴─┘	C9
क्या आप मुझे बता सकते हैं कि पिछले साल में आपके परिवार की औसतन आमदानी कितनी रही होगी। (सिर्फ एक का जवाब दें।)	प्रतिमहीना	└─┴─┴─┴─┴─┴─┴─┴─┘	C10a
	प्रति वर्ष	└─┴─┴─┴─┴─┴─┴─┴─┘	C10b
	जवाब देने से इंकार	88	C10c

Behavioural Measurements

तंबाकू का प्रयोग

अब हम आपसे कुछ तम्बाकू संबंधित प्रश्न पूछने जा रहें है।

Question	जवाब	Code
क्या आप वर्तमान में किसी भी तंबाकू उत्पाद जैसे बीडी, सिगरेट, हुक्का पान, तंबाकू, आदि का सेवन करते हैं।	हाँ 1<br>नहीं 2 यदि ना तो *T8*	T1
क्या आप वर्तमान में धुएं वाले तंबाकू पदार्थो का रोज सेवन करते है।	हाँ 1<br>नहीं 2	T2
आप कितने साल के थे जब आपने पहली बार सिगरेट पी थी।	उम्र (साल)<br>पता नहीं 77 └─┴─┘ यदि पता है तो *T5a/T5aw*	T3
क्या आपको याद है कि ये कितनी पुरानी बात है।	प्रति वर्ष └─┴─┘ यदि पता है तो *T5a/T5aw*	T4a
	या प्रति महीना └─┴─┘ यदि पता है तो *T5a/T5aw*	T4b
	या हफ्तों में └─┴─┘	T4c
निम्नलिखित पदार्थो में से औसतन आप कितने पदार्थो का सेवन रोजाना/साप्ताहिक करते हैं। (अगर रोजाना से कम, साप्ताहिक का उत्तर पूछिए।)	रोजाना↓ साप्ताहिक↓	
	निर्मित बनी हुई सिगरेट └─┴─┴─┴─┘ └─┴─┴─┴─┘	T5a/T5aw
	हाथों द्वारा तैयार सिगरेट └─┴─┴─┴─┘ └─┴─┴─┴─┘	T5b/T5bw
	तंबाकू वाला हुक्का └─┴─┴─┴─┘ └─┴─┴─┴─┘	T5c/T5cw
	सिगार, cheroots, cigarillos └─┴─┴─┴─┘ └─┴─┴─┴─┘	T5d/T5dw
	शिशा या हुक्के की गिनती └─┴─┴─┴─┘ └─┴─┴─┴─┘	T5e/T5ew
	अन्य └─┴─┴─┴─┘ └─┴─┴─┴─┘<br>यदि कोई और तो *T5other*, नहीं तो *T6*	T5f/T5fw
	अन्य (बताएं) └─┴─┴─┴─┴─┴─┘	T5other/ T5otherw
क्या आपने पिछले 12 महीनों में सिगरेट छोड़ने की कोशिश की है।	हाँ 1<br>नहीं 2	T6
पिछले 12 महीनों में क्या कभी किसी डॉक्टर या स्वास्थ्य कर्मचारी ने आपको तंबाकू छोड़ने की सलाह दी है।	हाँ 1 यदि *T2* =हाँ तब *T12*; यदि *T2*=ना तब *T9*<br>नहीं 2 यदि *T2* =हाँ तब *T12*; यदि *T2*= ना तब *T9*<br>पिछले 12 महीनों के दौरान कोई यात्रा नहीं 3 यदि *T2* =हाँ तब *T12*; यदि *T2*=ना तब *T9*	T7
अतीत में क्या आप कभी भी तंबाकू धुए का सेवन रोजाना करते थे।	हाँ 1<br>नहीं 2 यदि ना तो *T12*	T8
पिछले समय के दौरान आपने रोज़ सिगरेट पी है।	हाँ 1 यदि *T1*= हाँ तो *T12*, नहीं तो *T10*<br>नहीं 2 यदि *T1*= हाँ तो *T12*, नहीं तो *T10*	T9

आप कितने वर्ष के थे जब आपने सिगरेट पीना छोड़ दिया था।	उम्र (साल)	⌊__⌊__⌋ यदि पता है तो *T12*	T10
	पता नहीं *77*		
आपने कितने समय पहले सिगरेट पीना बंद किया।	साल पहले	⌊__⌊__⌋ यदि पता है तो *T12*	T11a
	या महीने पहले	⌊__⌊__⌋ यदि पता है तो *T12*	T11b
	या हफते पहले	⌊__⌊__⌋	T11c
क्या आप मौजूदा समय में धुआ रहित पदार्थो का सेवन कर रहे है तंबाकू, जरदा, खैनी आदि।	हाँ	1	T2
	नही	2 यदि ना तो *T15*	
मौजूदा समय में क्या आप रोजाना धुआ रहित तंबाकू पदार्थो का प्रयोग करते हैं।	हाँ	1	T13
	नही	2 यदि ना तो *T14aw*	
औसतन आप एक दिन हफ्ते में कितनी बार इस्तेमाल करते हो।<br>....<br>*(यदि एक रोज से कम है तो साप्ताहिक)*<br>*(RECORD FOR EACH TYPE, USE SHOWCARD)*<br>पता नहीं *7777*<br>*v*	रोजाना↓ साप्ताहिक↓		
	मुह के रास्ते तंबाकू लेना	⌊__⌊__⌊__⌊__⌋ ⌊__⌊__⌊__⌊__⌋	T14a/ T14aw
	नाक के रास्ते सूघना	⌊__⌊__⌊__⌊__⌋ ⌊__⌊__⌊__⌊__⌋	T14b/ T14bw
	तंबाकू का चबाना	⌊__⌊__⌊__⌊__⌋ ⌊__⌊__⌊__⌊__⌋	T14c/ T14cw
	सुपारी, तंबाकू	⌊__⌊__⌊__⌊__⌋ ⌊__⌊__⌊__⌊__⌋	T14d/ T14dw
	अन्य	⌊__⌊__⌊__ __⌊__⌊__⌋<br>यदि *T13* =ना है तो *T12*, नहीं तो *T17*	T14other/ T14other w
बीते हुए समय में क्या आपने धुआ रहित तंबाकू का प्रयोग किया है(तंबाकू, जर्दा, खैनी आदि।)	हाँ	1	T15
	नही	2 यदि ना तो *T17*	
बीते समय में क्या आपने धुआ रहित तंबाकू का प्रयोग रोजाना किया है। (गुटखा, जर्दा, खैनी आदि)	हाँ	1	T16
	नही	2	
पिछल 30 दिनो में क्या आपके घर में किसी ने सिगरेट/बीड़ी का सेवन किया है।	हाँ	1	T17
	नही	2	
पिछले 30 दिनों में क्या आपके काम करने वाले स्थान के आस-पास (उस इमारत या अन्य स्थल) किसी ने सिगरेट/बीड़ी का सेवन किया है।	हाँ	1	T18
	नही	2	
	अपनी नीजी सम्बन्धी क्षेत्र में कार्य ना करना	3	

शराब का सेवन

शराब का सेवन संबंधी पूछे गए प्रश्न

प्रश्न	उत्तर		Code
क्या आपने कभी भी शराब जैसे बीयर, वाईन या लोकल शराब का सेवन किया है ?	हाँ	1	A1
	नही	2 यदि ना तो *A16*	
क्या पिछले 12 महीनों में आपने किसी भी प्रकार की शराब का सेवन किया है?	हाँ	1 यदि हाँ तो *A4*	A2
	नही	2	
क्या आपने शराब का सेवन सेहत के लिए जिससे कि सेहत पर नकारात्मक प्रभाव या किसी डॉक्टर/स्वास्थ्य कर्मचारी की सलाह के कारण छोड़ दिया है?	हाँ	1 यदि हाँ तो *A16*	A3
	नही	2 यदि ना तो *A16*	
पिछले 12 महीनों के दौरान आपने कितनी बार लगातार कम से कम एक मानक शराब का सेवन किया है? *(READ RESPONSES, USE SHOWCARD)*	हर रोज	1	A4
	हफते के 5-6 दिन	2	
	हफते के 3-4 दिन	3	
	हफते के 1-2दिन	4	
	महीने के 1-3 दिन	5	
	एक महीने से कम	6	
पिछले 30 दिनों में आपने किसी शराब का सेवन किया है?	हाँ	1	A5
	नही	2 यदि ना तो *A13*	
पिछले 30 दिनों में कितने मौकों पर आपने कम से कम एक मानक शराब का सेवन किया है।	संख्या<br>पता नहीं *77*	└──┴──┘	A6
पिछले 30 दिनों में जब आपने शराब का सेवन किया था तब आपने औसतन कितनी मानक शराब का सेवन किया था। एक मौके पर? *(USE SHOWCARD)*	संख्या<br>पता नहीं *77*	└──┴──┘	A7
पिछले 30 दिनों में किसी एक मौके पर मानक शराब की सबसे बड़ी संख्या क्या थी सभी प्रकार की शराब को मिलाकर।	सबसे बड़ी संख्या<br>पता नहीं *77*	└──┴──┘	A8
पिछले 30 दिनों में आपने 6 या इससे अधिक मानक शराब का सेवन कितनी बार किया है किसी शराब पीने के मौके पर।	कितनी बार<br>पता नहीं *77*	└──┴──┘	A9
पिछले 7 दिनों में आपने कितनी मानक शराब हर रोज ली है। *(USE SHOWCARD)* पता नहीं *77*	सोमवार	└──┴──┘	A10a
	मंगलवार	└──┴──┘	A10b
	बुधवार	└──┴──┘	A10c
	वीरवार	└──┴──┘	A10d
	शुक्रवार	└──┴──┘	A10e
	शनिवार	└──┴──┘	A10f
	रविवार	└──┴──┘	A10g

मैं सिर्फ आपको आपके पिछले 7 दिनों के दौरान किये गये शराब के सेवन के बारे में पूछा है आमतौर पर यह प्रश्न शराब के बारे में है जो कि अगले प्रश्न आपके शराब के सेवन से संबधित है आप की निकली हुई शराब, सीमा पार से लाई गई शराब या दूसरे देश से लाई गई शराब बिना मकस्द से पी इसके इलावा आप शराब या अन्य कोई लावारिस शराब पी रहे हो, कृप्या करके उत्तर देते समय इस तरह की शराब के बारे में ही सोचिये।

प्रश्न	उत्तर		Code
पिछले 7 दिनों में क्या आपने घर की निकली हुई शराब, या सीमा पार से लाई गई, या दूसरे देश की शराब का सेवन किया है। *(USE SHOWCARD)*	हाँ	1	A11
	नही	2 यदि ना तो *A13*	
औसतत पिछले 7 दिनों में आपने इनमें से कितनी मानक शराब का नियमित रूप से सेवन किया है। *(USE SHOWCARD)* *Don't Know 77*	घर की तैयार देसी शराब	└─┴─┘	A12a
	घर की तैयार बीयर या शराब	└─┴─┘	A12b
	घर की तैयार शराब, बीयर	└─┴─┘	A12c
	शराब जो सेवन के लिए नहीं बनी है जैसे दवाईयां, इत्र आदि।	└─┴─┘	A12d
	देश की कोई अन्य untaxed शराब	└─┴─┘	A12e
पिछले 12 महीनों में आपने कितनी बार महसूस किया है। कि शराब पीना शुरू करने के बाद आप उसे छोड़ने में दिक्कत महसूस कर रहे हैं?	ज्यादातर	1	A13
	साप्ताहिक	2	
	मासिक	3	
	एक महीने से घट	4	
	कभी नहीं	5	
पिछले 12 महीनों में शराब पीने के कारण कितनी बार आपने काम करने की कोशिश करी पर उसमें असफल रहे?	रोजाना या ज्यादातर	1	A14
	साप्ताहिक	2	
	मासिक	3	
	एक महीने से घट	4	
	कभी नहीं	5	
पिछले 12 महीनों में रात को ज्यादा शराब पीने के बाद आपको सुबह उठते ही कितनी बार शराब की जरूरत महसूस हुई है।	रोज़ या हर रोज़	1	A15
	हफ्ते में	2	
	महीने में	3	
	एक महीने से घट	4	
	कभी नहीं	5	
पिछले 12 महीनों में कभी किसी और की पी हुई शराब के कारण आपको किसी परिवारिक मुश्किल या आपके जीवन साथी के साथ कोई दिक्कत आई है।	हाँ जी, महीने से ज्यादा	1	A16
	हाँ जी महीने में	2	
	कई बार, पर महीनों में घट	3	
	हाँ जी , एक या दो बार	4	
	नहीं	5	

खुराक		
अगले प्रश्न फल और सब्जियों से संबंधित जो आप रोजाना खाते हैं। मेरे पास एक पोषण कार्ड है जो कि उदाहरणमय रूप से फल और सब्जियों का बताएगा। हर एक चित्र खाने का नाम दर्शाएगा। जब आप जवाब दें तो पिछले साल के महत्वपूर्ण एक हफते को ध्यान में रखते हुए इन प्रश्नों का जवाब दें।		
प्रश्न	उत्तर	Code
आप एक हफ्ते में कितने दिन फलों का सेवन करते हैं? *(USE SHOWCARD)*	दिनों की संख्या └─┴─┘<br>पता नहीं 77<br>यदि एक भी दिन नहीं तो D3	D1
और इनमें से किसी एक दिन आप कितनें फलों का सेवन करते हैं?	servings की संख्या └─┴─┘<br>पता नहीं 77	D2
आप एक हफ्ते में कितने दिन सब्जियाँ खाते हो? *(USE SHOWCARD)*	दिनों की संख्या └─┴─┘<br>पता नहीं 77<br>यदि एक भी दिन नहीं तो D3	D3
किसी एक हफ्ते में, उन दिनों में से किसी एक दिन आप कितनी सब्जियों का सेवन करते हैं।	servings की संख्या └─┴─┘<br>पता नहीं 77	D4

Dietary Salt		
अगले प्रश्न में हम आपसे आपके खाने में खाने वाले नमक कच्चा नमक जैसे कि खारा नमक आयोजित मुक्त नमक और चूरन या नमकीन चटनियाँ जैसे कि सोया, हरी, चटनी, मिर्च, की चटनी आम का आचार आने वाले अगले प्रश्नों में हम आपसे खाना बनाते समय खाते हुए अथवा अचार या नमक से तैयार होने वाले खाने वाली चीजें के सेवन को काबू करने के बारे में पूछा जा रहा है। आप इन प्रश्नों के जवाब तब दें अगर आपको लगता है कि आप नमक का कम सेवन करते है।		
प्रश्न	उत्तर	Code
आप कितनी बार अपने खाने में उपर से नमक या नमकीन चटनी मिलाते हैं? (केवल एक ही चुनें) *(USE SHOWCARD)*	हर समय 1<br>अकसर 2<br>कभी-कभी 3<br>बहुत कम 4<br>कभी नहीं 5<br>पता नहीं 77	D5
आपके घर में कितनी बार खाना पकाते हुए उसमें नमक या नमकीन चटनी मिलाई जाती है।	हर समय 1<br>अकसर 2<br>कभी-कभी 3<br>बहुत कम 4<br>कभी नहीं 5<br>पता नहीं 77	D6
आप कितनी बना हुआ खाना जिसमें नमक की मात्रा अधिक होती है। उनका सेवन करते हैं। बना हुआ खाना, नमकीन सनैकस, बंद भोजन फास्ट फूड आदि?	हर समय 1<br>अकसर 2<br>कभी-कभी 3<br>बहुत कम 4<br>कभी नहीं 5<br>पता नहीं 77	D7
आपके अनुसार आप कितना नमक या नमकीन चटनी का प्रयोग करते हो।	बहुत ही ज्यादा 1<br>बहुत ज्यादा 2<br>Normal मात्रा/सही 3<br>बहुत कम 4<br>बहुत ही कम 5	D8

	पता नहीं 77	
आप अपने खाने में नमक की मात्रा कम करना कितना महत्वपूर्ण मानते/समझते हैं?	बहुत जरूरी 1<br>सिर्फ जरूरत पर 2<br>जरूरी नहीं 3<br>पता नहीं 77	D9
क्या आपको लगता है कि बहुत ज्यादा नमक या नमकीन चटनी आपकी सेहत को खराब करने का कारण हो सकती है।	हाँ 1<br>नहीं 2<br>पता नहीं 77	D10
नमक के सेवन को काबू करने के लिये आप इनमें से आप रोज़ाना क्या कुछ कर रहे हो।<br>*(RECORD FOR EACH)*		
पक्के हुए भोजन के सेवन की सीमा	हाँ 1<br>नहीं 2	D11a
भोजन के लेबल पर नमक या सोडियम को मात्रा को देखते हुये।	हाँ 1<br>नहीं 2	D11b
क्या आप कम सोडियम युक्त नमक को खरीदते है।	हाँ 1<br>नहीं 2	D11c
खाना बनाते समय नमक के इलावा अन्य मसालों का प्रयोग करते हो।	हाँ 1<br>नहीं 2	D11d
घर से बाहर तैयार किये गये भोजन के सेवन को अनदेखा करते हो।	हाँ 1<br>नहीं 2	D11e
कुछ अन्य चीजें जो कि खासतौर पर नमक के सेवन पर काबू करे	हाँ 1 यदि हाँ तो *D11other*<br>नहीं 2	D11f
अन्य (कृपया बताएं)	|__|__|__|__|__|__|__|	D11other

भोजन बनाने के लिए आपके घर में किस तरह का तेल या घी का प्रयोग करते हैं।<br>*(USE SHOWCARD)*<br>*(SELECT ONLY ONE)*	सरसों का तेल	1	D12
	सोयाबीन तेल	2	
	मक्खन या घी	3	
	सुरजमुखी का तेल	4	
	और	5 यदि अन्य तो *D12 other*	
	इनमें से कोई नहीं	6	
	कोई भी उपयोग नहीं	7	
	पता नहीं	77	
	अन्य	|__|__|__|__|__|__|__|	D12other
औसतन आप एक हफ्ते में कितना भोजन खाते हो जो कि बाहर तैयार किया जाता है भोजन से मतलब है नाश्ता, दोपहर का खाना, रात का खाना	संख्या<br>पता नहीं 77	|__|__|	D13

<table>
<tr><th colspan="3">Physical Activity</th></tr>
<tr><td colspan="3">आगे में अब आपके खास तौर पर आपने एक हफ्ते में आपने किस तरह की शारीरिक गतिविधियाँ कर समय व्यतीत करते हो के बारे में पूछने जा रहा हूँ कृपया करके आप इसका जवाब दें। चाहे बेशक आप अपने आप को शारीरिक तौर पर तंदरूसत नहीं मानते।
सबसे पहले आप काम करते समय व्यतीत करने के बारे में सोचो। जो चीजें आपके पास काम करने के बारे में सोचें जो कि आप पैसे ले के या बिना पैसों के करते हो जैसे कि पढ़ाई, सिखाई, फसलों की कटाई, मछली पालन या भोजन के लिये शिकार करना, रोज़गार की तलाश।</td></tr>
<tr><th>प्रश्न</th><th>उत्तर</th><th>Code</th></tr>
<tr><th colspan="3">Work</th></tr>
<tr><td>क्या आप जोर वाले काम को कम से कम दस मिनट करते हो जिसके कारण आपकी सांस फूलती है। या आपके दिल की धड़कन बढ़ जाती है
(USE SHOWCARD)</td><td>हाँ 1
नहीं 2 यदि ना तो P 4</td><td>P1</td></tr>
<tr><td>आमतौर पर एक हफ्ते में काम करने के दौरान कितने दिन ज्यादा जोर देने वाले काम को करते हो।</td><td>दिनों की संख्या ⎿_⏌</td><td>P2</td></tr>
<tr><td>आमतौर पर एक दिन में आप अपने काम के दौरान कितने समय जोर वाले काम को देते हैं।</td><td>Hours : minutes ⎿_⎿_⏌ : ⎿_⎿_⏌
hrsmins</td><td>P3
(a-b)</td></tr>
<tr><td>क्या आप कम से कम 10 मिनट लगातार कमजोर वाले काम कर लेते हो। जिससे आपको कम सांस चढ़ता है दिल की धड़कन कम बढ़ती है जैसे कि हल्का भार उठाना, हाथ से कपड़े धोना झाड़ू मारना, पोचा मारना कुएं से पानी निकालना हाथ से कुछ पीसना बाजार से करियाने का सामान खरीदना (USE SHOWCARD)</td><td>हाँ 1
नहीं 2 यदि ना तो P 7</td><td>P4</td></tr>
<tr><td>एक हफते में काम करने के दौरान आप कितने दिन जोर वाले काम करते है।</td><td>दिनों की संख्या ⎿_⏌</td><td>P5</td></tr>
<tr><td>आमतौर पर एक दिन में आप अपने काम के दौरान कितना समय कमजोर वाले काम करते हैं।</td><td>Hours / Minutes ⎿_⏌ ⎿_⏌</td><td>P6</td></tr>
<tr><th colspan="3">Travel to and from places</th></tr>
<tr><td colspan="3">अगला प्रश्न काम करने के दौरान शारीरिक गतिविधियों को छोड़कर है इस का जिक्र पहले हो चुका है। अब मैं आपसे आपके आम रास्ते के सफर, किसी जगह से किसी जगह तक का सफर के बारे में पूछा जा रहा है। जैसे कि उदाहरण के तौर पर काम से खरीदारी लिये, बाज़ार के लिये तथा माथा टेकने वाली जगह तक जाने के लिये</td></tr>
<tr><td>क्या आप कम से कम 10 मिनट लगातार पैदल या साईकिल के प्रयोग से एक स्थान से दूसरे स्थान तक जाते हैं।</td><td>हाँ 1
नहीं 2 यदि ना तो P 10</td><td>P7</td></tr>
<tr><td>आप एक हफ्ते में कितने दिन कम से कम 10 मिनट लगातार पैदल या साईकिल के प्रयोग से एक स्थान से दूसरे स्थान जाते है।</td><td>दिनों की संख्या ⎿_⏌</td><td>P8</td></tr>
<tr><td>आमतौर पर आप एकदिन में कितना पैदल या साईकल से सफर करते हैं।</td><td>Hours : minutes ⎿_⎿_⏌ : ⎿_⎿_⏌
hrsmins</td><td>P9
(a-b)</td></tr>
</table>

मनोरंजन गतिविधियाँ		
अगला प्रश्न काम और यातायात की गतिविधियों को छोड़ के है क्योंकि इसका जिक्र पहले हो चुका हैं। अब मैं आपसे खेलों से संबधित, सेहत से संबधित और मनोरंज़न गतिविधियाँ के संबध में आपसे पूछा जा रहा है।		
क्या आप लगातार 10 मिनट खेलने कसरत करने या मनोरंजन या जोर लगने वाली गतिविधियां करते हो।<br>जिससे आपका ज्यादा दम फूलता है या दिल की धड़कन तेज होती है। *(USE SHOWCARD)*	हाँ 1<br>नहीं 2 यदि ना तो *P 13*	P10
आमतौर पर आप एक हफते में कितने दिन ज्यादा जोर वाली गतिविधियां करते हो। (खेल, कसरत आदि।)	दिनों की संख्या └─┘	P11
आप एक दिन में कितने समय ज्यादा ज़ोर वाली गतिविधियां करते हो। (खेल, कसरत, मनोरंजन संबंधी।)	Hours : minutes └─┴─┘ : └─┴─┘<br>hrsmins	P12 (a-b)
क्या आप लगातार 10 मिनट खेल कसरत, या मनोरंजन या हल्की जोर वाली गतिविधियां करते हो। (साइकलिंग, तैराकी, वालीबॉल)<br>*(USE SHOWCARD)*	हाँ 1<br>नहीं 2 यदि ना तो *P16*	P13
आप एक हफते में कितने दिन कितने समय हल्के ज़ोर वाली गतिविधियां करते हो।	दिनों की संख्या └─┘	P14
आप एक दिन में कितना समय हल्के जोर वाली गतिविधियां करते हो।<br>(खेल, कसरत, मनोरंजन संबंधी।)	Hours : minutes └─┴─┘ : └─┴─┘<br>hrsmins	P15 (a-b)

सुस्त व्यवहार			
निम्नलिखित प्रश्न जैसे कि बैठना उठना झुकना काम के दौरान या किसी एक स्थान से दूसरे स्थान तक, अपने दोस्तों के साथ समय बिताना बैठकर, या बस, गाड़ी, ट्रेन में यात्रा करना, पत्ते खेलना, टीवी देखना, लेकिन इसमें सोने के समय को ना लिया जाए			
आमतौर पर एक दिन में आप कितना समय बैठ के या सुस्ती आलस में गुजरते हो	Hours : minutes	└─┴─┘ : └─┴─┘<br>hrsmins	P16 (a-b)

बढ़े हुए खून के दबाव की पुरानी जानकारी		
**प्रश्न**	**उत्तर**	**Code**
क्या आपने कभी किसी डॉकटर या स्वास्थ्य कर्मचारी से अपने रक्ताचाप की जांच करवाई है?	हाँ 1<br>नहीं 2 यदि ना तो *H6*	H1
क्या कभी किसी डॉक्टर या स्वास्थ्य कर्मचारी ने बताया है कि आपका ब्लड प्रेशर बढ़ा हुआ है।	हाँ 1<br>नहीं 2 यदि ना तो *H6*	H2a
पिछले 12 महीनों में किसी ने बताया है आपको	हाँ 1<br>नहीं 2	H2b
पिछले 2 हफ्तों में बढ़े हुए रक्तचाप/ब्लड प्रैशर के लिए क्या आपने किसी डॉक्टर या स्वास्थ्य कर्मचारी द्वारा निर्धारित दवाई का	हाँ 1<br>नहीं 2	H3

सेवन किया है।।		
क्या आपने कभी बढ़े हुए ब्लड प्रैशर या उच्च रक्तचाप के लिये पारंपरिक आरोग्य दवाइयों का सेवन किया है।	हाँ 1<br>नहीं 2	H4
मौजुदा समय में क्या आपने बढ़े हुए कैल्सट्रोल के लिए कोई हर्बल या पारम्परिक वस्तु का प्रयोक कर रहें हैं?	हाँ 1<br>नहीं 2	H5

शुगर की जानकारी		
क्या आपने कभी अपने ब्लड शुगर की जांच डॉक्टर या स्वास्थ्य कर्मचारी से करवाई है?	हाँ 1<br>नहीं 2 यदि ना तो *H12*	H6
क्या कभी किसी डॉक्टर या स्वास्थ्य कर्मचारी ने आपको आपके खून में बढ़ रही शुगर या मधुमेह के बारे में बताया है?	हाँ 1<br>नहीं 2 यदि ना तो *H12*	H7a
पिछले 12 महीनों में आपकी शूगर के बारे में बताया गया है।	हाँ 1<br>नहीं 2	H7b
पिछले दो हफतों में मधुमेह के लिए क्या आपने किसी डॉक्टर या स्वास्थ्य कर्मचारी द्वारा निर्धारित की गई दवाई का सेवन किया है।	हाँ 1<br>नहीं 2	H8
क्या मौजूदा समय में आप डॉक्टर या स्वास्थ्य कर्मचारी द्वारा निर्धारित मधुमेह के लिए इन्सूलिन ले रहे हैं?	हाँ 1<br>नहीं 2	H9
क्या आपने कभी अपने बढ़ते हुए शूगर के लिए पारम्परिक आरोग्य दवाईयों का सेवन किया है।	हाँ 1<br>नहीं 2	H10
मौजूदा समय में क्या आप इस समय अपने शुगर के लिए किसी हर्बल या पारंपरिक दवाई का सेवन कर रहे हैं।	हाँ 1<br>नहीं 2	H11

कैल्सटरोल की पुरानी जानकारी		
**Question**	**Response**	**Code**
क्या आपने कभी अपने कैलोस्ट्रोल की जांच किसी डॉक्टर या स्वास्थ्य कर्मचारी से करवाई है।	हाँ 1<br>नहीं 2 यदि ना तो *H17*	H12
क्या आपको कभी आपके बढ़े हुए कैलोस्ट्रोल के बारे में डॉक्टर या स्वास्थ्य कर्मचारी ने बताया है।	हाँ 1<br>नहीं 2 यदि ना तो *H17*	H13a
क्या पिछले 12 महीनों में बताया गया है?	हाँ 1<br>नहीं 2	H13b
क्या पिछले दो हफतों में आपने बढ़े हुए कैलोस्ट्रोल के लिए किसी डॉक्टर या स्वास्थ्य कर्मचारी द्वारा निर्धारित दवाई का सेवन मौखिक रूप से किया है।	हाँ 1<br>नहीं 2	H14
क्या आपने कभी अपने बढ़े हुए कैलस्ट्रोल के लिए किसी पारंपरिक आरोग्य केंद्र में दिखवाया है।	हाँ 1<br>नहीं 2	H15
क्या आपने कभी हर्बल या पारम्परिक नुस्खे अपनाये हैं। बढ़े हुए कैल्सटरोल के लिए।	हाँ 1<br>नहीं 2	H16

दिल की बीमारी की पुरानी जानकारी			
क्या आपको कभी हृदय रोग या आघात के कारण दिल का दौरा या सीने में दर्द हुआ है।	हाँ<br>नहीं	1<br>2	H17
मौजूदा समय में क्या आप हृदय रोगे रोकने या दूर करने के लिए एसपरीन का सेवन कर रहे हैं?	हाँ<br>नहीं	1<br>2	H18
मौजूदा समय में क्या आप हृदय रोगे रोकने या दूर करने के लिए स्टैटिन (lovastatin/sunvastatin/atorvaspatin) का सेवन कर रहे हैं नियमित तौर पर	हाँ<br>नहीं	1<br>2	H19

पथरी की बीमारी की पुरानी जानकारी			
क्या कभी आपको किसी डॉक्टर या स्वास्थ्य कर्मचारी द्वारा बताया गया है कि आपके गुर्दे में पत्थरी है।	हाँ<br>नहीं	1<br>2	HX1
क्या कभी आपको किसी डॉक्टर या स्वास्थ्य कर्मचारी द्वारा अपको बताया गया है कि आपके कमजोर गुर्दे के बारे में बताया गया है।	हाँ<br>नहीं	1<br>2	HX2
क्या आप कभी डेलिसिस पर रहें हैं या आपने गुर्दे बदलवाएं हैं।	हाँ<br>नहीं	1<br>2	HX3

जीवन व्यतीत करने की सलाह			
पिछले तीन वर्षों के उपरांत क्या किसी डॉक्टर या स्वास्थ्य कर्मचारी ने आपको निम्नलिखित में से किसी के लिए सलाह दी (RECORD FOR EACH)			
तंबाकू का प्रयोग न करो या न शुरू करो।	हाँ<br>नहीं	1<br>2	H20a
खुराक में नमक को कम करो।	हाँ<br>नहीं	1<br>2	H20b
दिन में फल और सब्जियां का घट से घट पांच बार सेवन करो।	हाँ<br>नहीं	1<br>2	H20c
खाने में तेल, घी को कम करो।	हाँ<br>नहीं	1<br>2	H20d
शारीरिक गतिविधियां शुरू करो।	हाँ<br>नहीं	1<br>2	H20e
अपने शरीर को संतुलित रखो या वजन कम करें।	हाँ<br>नहीं	1 यदि *C1=2* है तो *CX1*<br>2	H20f

Health screening

गर्भाशय का कैंसर

अगला प्रश्न बच्चेदानी के कैंसर से बचाव के लिये पूछे जा रहे हैं बच्चेदानी के कैंसर से बचाव के लिये इसकी जांच कई तकनीकों से की जाती है। Vishudl inspection with Acitic Acid ऐसिड के साथ Pap smear और Human Papillomavirus (HPV) test के द्वारा जांच । इसमें जांच के लिये यूटरीन सविकास के तल से ऐसिड लगा दिया जाता है। इन दोनों तरीकों के लिये डाक्टर या नर्स रूई के टुकड़े के ऊपर योनि के बीच का पदार्थ लेते हैं। और जाँच के लिये लैबरोटरी भेज देते हैं। यहां पर यह संभव है कि आप भी रूई के टुकड़े को योनि में रख सकते हो। लैबरोटरी में Pep samiaar टेस्ट के दौरान बच्चेदानी में असाधारण सैल की जाँच की जाती है और H-P-V की जांच के समय विषाणु की जांच की जाती है।

प्रश्न	उत्तर	कोड
**यह सवाल केवल महिलाओं के लिए हैं?**		

क्या कभी आपने गर्भाशय कैंसर की जाँच ऊपर दिए गए किसी तरीके से करवाई है?	हाँ 1<br>नहीं 2<br>पता नहीं 77	CX1

**स्तन का कैंसर**			
**निम्नलिखित प्रश्न X1 - X2 केवल महिलाओं के लिए है?**			
क्या आपको कभी किसी ने स्तन के कैंसर के स्तन की जांच करना सिखवाया या दिखवाया गया है।	हाँ<br>नहीं	1<br>2	X1
आपने आखिरी समय अपने स्तन की जाँच कब करवाई थी?	एक साल या घट<br>एक साल या दो सालों के<br>दो सालों से ज्यादा<br>कभी नहीं<br>पता नहीं	1<br>2<br>3<br>4<br>77	X2
आपने आखिरी बार अपना मैमोग्राम कब करवाया था।	एक साल या घट<br>एक साल या दो सालों के<br>दो सालों से ज्यादा<br>कभी नहीं<br>पता नहीं	1<br>2<br>3<br>4<br>77	X3
**(मौखिक) जुबान का कैंसर**			
क्या आपने कभी अपने मुंह की परत या जीभ की जांच किसी दन्त चिकित्सक या डॉक्टर द्वारा करवाई है?	हाँ	1	X4
	नहीं	2	
	पता नहीं	77	
	जवाब देने से इंकार	88	88
हाल ही में आपके मौखिक कैंसर की जांच कब हुई है?	एक साल या घट	1	X5
	एक साल या दो सालों के	2	
	दो सालों से ज्यादा	3	
	कभी नहीं	4	
	पता नहीं	77	

परिवार की पुरानी जानकारी		
**प्रश्न**	**उत्तर**	**कोड**
क्या आपके परिवार का कोई सदस्य इनमें से किसी बिमारियों से पीड़ित है।		
मधुमेह या खून में बढ़ी हुई शूगर	हाँ 1<br>नहीं 2	F1A
उच्च रकतचाप	हाँ 1<br>नहीं 2	F1B
झटका/आघात	हाँ 1<br>नहीं 2	F1C
कैंसर या रसौली/घातक फोड़ा	हाँ 1<br>नहीं 2	F1D
बढ़ा हुआ कैल्सटरोल	हाँ 1<br>नहीं 2	F1E
दिल का दौरा(पुरूषों मे 55 साल से कम और औरतों में 65 साल से कम)	हाँ 1<br>नहीं 2	F1f
पुरानी गुर्दे की बीमारी	हाँ 1<br>नहीं 2	F1g

दिमागी संतुलन (Mental Health)		
पिछले 12 महीनों में क्या आपने आत्महत्या करने के बारे में विचार किया?	हाँ 1<br>नहीं 2<br>जवाब देने से इंकार 3	MH1
क्या आपने ऐसी सोच के लिए किसी व्यवसायिक व्यक्ति की सहायता ली है।	हाँ 1<br>नहीं 2<br>जवाब देने से इंकार 3	MH2
पिछले 12 महीनों में क्या आपने ऐसा सोचा है कि आत्महत्या कैसे करनी है।	हाँ 1<br>नहीं 2<br>जवाब देने से इंकार 3	MH3
क्या आपने कभी आत्महत्या करने की कोशिश की है।	हाँ 1<br>नहीं 2<br>जवाब देने से इंकार 3	MH4
पिछले 12 महीनों आपने आत्मदाह करने की कोशिश की है।	हाँ 1<br>नहीं 2<br>जवाब देने से इंकार 3	MH5

पिछली बार आत्महत्या करने की कोशिश में कौन सा तरीका अपनाया था?	चाकू या कोई नुकीला औज़ार 1<br>अधिक मात्रा में दवाई<br>(जो निर्धारित की गई हो काउंटर से) 2<br>किसी अन्य पदार्थ की अधिक मात्रा<br>(जैसे हीरोईन या शराब आदि) 3<br>कीटनाशक जहर (जैसे चूहे मारने की दवा, 4<br>कीटनाशक या वनस्पति नाशक)<br>अन्य जहर (जैसे पौधे बीज परिवार उत्पाद) 5<br>जहरीली गैसें कोयले से निकली हुई 6<br>अन्य 7 यदि अन्य तो *MH6 other*<br>नकार देना 88	MH6
	अन्य (निर्दिष्ट) |__|__|__|__|__|__|__|	MH6 other
क्या आपने इस कोशिश के लिये मैडिकल सहायता ली है	हाँ 1<br>नहीं 2<br>जवाब देने से इंकार 3	MH7
आप इस कोशिश के कारण किसी अस्पताल में दाखिल हुए हैं।	हाँ 1<br>नहीं 2<br>जवाब देने से इंकार 3	MH8
क्या आपके किसी सगे-संबंधी में किसी ने आत्मदाह करने की कोशिश की है।	हाँ 1<br>नहीं 2<br>जवाब देने से इंकार 3	MH9
क्या आपके किसी करीबी सगे-संबंधी की मृत्यु आत्महत्या करने से हुई है?	हाँ 1<br>नहीं 2<br>जवाब देने से इंकार 3	MH10

**स्वास्थय देख-रेख**		
आगें मैं आपकी सेहत बीमा कवरेज़ और आपको हुई किसी भी गैरसंचारी रोग के संबंध में सेहत सेवाओं के प्रयोग के बारे में पूछने जा रहा हूँ गैरसंचारी रोगों में हदय रोग। कैंसर, फेफडो की बीमारी। जैसे कि दमा, फेफड़ों की बीमारीयों, फेफडों में उच्च रकतचाप के कारण सांस की बीमारी और मधुेह शामिल है।		
**Health Care Coverage**		
आपकी मौजूदा सेहत बीमा कवरेज़ के बारे में जानकारी दो। सेहत बीमा कवरेज का मतलब है कि उपरांत सेहत संभाल के खर्चे के लिये आपको अदायगी करता है।		
क्या आपका इस समय सेहत का बीमा है।	हाँ 1	HC1

	नहीं	2 यदि ना तो *HC3*	
आपके पास इस समय किस प्रकार का सेहत का बीमा है	मेन्डेटरी हैल्थ इंश्योरेंश प्लान<br>वोलेन्टरी हैल्थ इंश्योरेंश प्लान<br>अन्य<br>पता नहीं	1<br>2<br>3 यदि अन्य तो *HC2other*<br>77	HC2
	यदि अन्य (बताइये)	________	HC2 other
पिछले 12 महीनों में निम्नलिखित वित्तीय स्त्रोत में से आपने अपने स्वास्थ्य से जुड़े भुगतान जैसे दवाईयां उपचार सलाह इलाज के लिए किसका प्रयोग किया?	परिवार की जूदा आमदल	हाँ 1<br>नहीं 2	HC3a
	बचत	हाँ 1<br>नहीं 2	HC3b
	सेहत बीमा भुगतान अदासगी	हाँ 1<br>नहीं 2	HC3c
	इकाई की बिक्री (उदाहरण फर्नीचर, जेवर, जानवर)	हाँ 1<br>नहीं 2	HC3d
	परिवार के करीबी या घर से बाहर दोस्त	हाँ 1<br>नहीं 2	HC3e
	परिवार के इलावा और किसी के पास उधारे	हाँ 1<br>नहीं 2	HC3f
	और अन्य	हाँ 1 यदि अन्य तो *HC3other*<br>नहीं 2	HC3g
	कृपया निर्धारित करो।	________	HC3 other
**सेहत संभाल उपयोगता**			
**आपने किसी भी सेहत केन्द्र का दौरा करने और वहाँ प्राप्त उपचार के बारे में साचो। जो कि आपको हुई किसी गैरसचारी बीमारियों से संबधित हैं**			
क्या आपको कभी भी कोई गैरसंचारी बीमारी जैसे उदाहरण दिल का रोग सांस की बीमारी, डायबिटिज थी या है।	हाँ<br>नहीं	1<br>2 यदि ना तो *[next section]*	HC4
	यदि हाँ तो कृपया एन.सी.डी का नाम बताईये		HC4X
क्या आपने कभी भी किसी गैरसंचारी बीमारी के लिये सेहत केन्द्र का दौरा किया	हाँ<br>नहीं	1<br>2 यदि ना तो *HC11*	HC5
पिछली बार स्वास्थ्य केंद्र के दौरे में आपने वहां आने और जाने में कुल मिलाकर कितना समय व्यतीत किया?	Days : hours : minutes	____ : ____ : ____<br>days hrsmins	HC6
आखिरी बार जब आप स्वास्थ्य केंद्र गए तब डॉक्टर को दिखाने से पहले आपको कितना इंतजार करना पड़ा?	Hours : minutes	____ : ____<br>hrsmins	HC7
पिछले 30 दिनों के दौरान क्या आपने गैरसंचारी बीमारी के कारण किसी स्वास्थय केन्द्र का दौरा किया है।	हाँ<br>नहीं	1<br>2 यदि ना तो *HC11*	HC8

पछले 30 दिनों के दौरान गैरसंचारी बीमारी के कारण आपने कितनी बार स्वास्थ्य केन्द्र का दौरा किया।<br>(हर एक के लिए जवाब लिखें)<br>*(RECORD FOR EACH)*<br>*[INSERT COUNTRY-SPECIFIC CATEGORIES]*<br>*Don't know 77*	सेहत केन्द्र	____	HC9a
	जनता अस्पताल	____	HC9b
	निजी अस्पताल	____	HC9c
	डॉक्टर के दफतर	____	HC9d
	अन्य	____ यदि अन्य तो *HC9other*	HC9e

	अन्य (बताइये):	└┴┴┴┴┴┘	HC9otehr
पिछले 30 दिनों के दौरान किसी गैरसंचारी रोग के कारण स्वास्थ्य केंद्र के दौरे में आपका कुल कितना खर्चा हुआ।<br>*(RECORD FOR EACH OR PUT TOTAL AMOUNT)*<br>पता नहीं *77777*	सेहत सुरक्षा की फीस	└┴┴┴┴┘ [INR]	HC10a
	दवाईयाँ	└┴┴┴┴┘ [INR]	HC10b
	जांच	└┴┴┴┴┘ [INR]	HC10c
	यातायात	└┴┴┴┴┘ [INR]	HC10d
	अन्य	└┴┴┴┴┘ [INR]	HC10e
	और कुल राशी	└┴┴┴┴┘ [INR]	HC10f

पिछले 30 दिनों के दौरान आपने अपनी सेहत की देखभाल के लिये कितने खर्चा कर चुके हो जो कि आपके अस्पताल के खर्चे से संबधित हो जैसे कि रोज की दवाईयाँ<br>पता नहीं *77777*	राशी └┴┴┴┴┴┘ [INR]	HC11
पिछले 12 महीनों के दौरान क्या आप गैरसंचारी रोग के कारण अस्पताल में भर्ती हुए हैं।	हाँ 1<br>ना 2 *If No, go to HC15*	HC12
पिछले 12 महीनों के दौरान गैरसंचारी रोग के कारण आप कितने दिन अस्पताल में भर्ती रहे हैं।	दिनों की गिनती └┴┴┘	HC13
पिछले 12 महीनों के दौरान किसी गैरसंचारी रोग के कारण स्वास्थ्य केंद्र के दौरे में आपका कुल कितना खर्चा हुआ।<br>*(RECORD FOR EACH OR PUT TOTAL AMOUNT)*<br>*Don't know 77777*	सेहत सुविधाएं करने की फीस └┴┴┴┴┴┘ [INR]	HC14a
	दवाईयाँ └┴┴┴┴┴┘ [INR]	HC14b
	जाँच └┴┴┴┴┴┘ [INR]	HC14c
	यातायात └┴┴┴┴┴┘ [INR]	HC14d
	Other └┴┴┴┴┴┘ [INR]	HC14e
	अन्य कुल राशि └┴┴┴┴┴┘ [INR]	HC14f
**Home Care**		
कृपया करके आपके परिवार के सदस्य या दोस्तों द्वारा आपकी गैरसंचारी बीमारियों के कारण आपको दी गई घरेलू देखभाल के बारे में विचार करो।		
पिछले 30 दिनों के दौरान क्या आप किसी गैरसंचारी बीमारी के कारण आपके परिवार के किसी सदस्य ने आपको देखभाल की।	हाँ 1<br>ना 2 यदि ना तो *HC17*	HC15
पिछले 30 दिनों के दौरान उस सदस्य ने एक हफते में कितने घंटे आपकी देखभाल की है।<br>*Don't know 777*	Hours per week └┴┴┘ hrs	HC16
**Loss of Productivity**		
कृपया करके अपने उस समय के बारे में सोचो जब किसी गैरसंचारी रोग के कारण आपका कोई भी काम छूट गया हो। (उदाहरण के लिये काम, घर का काम, अध्ययन आदि कर पाये हों)		
पिछले 30 दिनों के दौरान क्या आप किसी गैर-संचारी रोग के कारण आप किसी भी काम के कारण छूट गये है।	हाँ 1<br>ना 2 यदि ना तो *[next section]*	HC17
पिछले 30 दिनों के दौरान क्या आप किसी गैर-संचारी बीमारी के कारण कितने दिन आपका काम छूटा है।	दिनों की संख्या └┴┘ दिन	HC18

Physical Measurements

खून का दबाव

प्रशन	उत्तर	Code
मुलाकात करता की पहचान	└┴┴┘	M1
रक्तचाप के लिए डिवाइस आई. डी	└┴┘	M2
कफ का आकार	छोटा, मध्यम , बड़ा	
पढ़ना 1	सिस्टोलिक(mmHg) └┴┴┘	M4a
	डायस्टोलिक (mmHg) └┴┴┘	M4b

पढ़ना 2	सिस्टोलिक (mmHg) |__|__|__|	M5a
	डायस्टोलिक (mmHg) |__|__|__|	M5b
पढ़ना 3	सिस्टोलिक (mmHg) |__|__|__|	M6a
	डायस्टोलिक (mmHg) |__|__|__|	M6b
पिछले दो हफतों के दौरान क्या आपके बढ़े हुए रक्तचाप के लिए किसी डॉक्टर या स्वास्थ्य कर्मचारी द्वारा इलाज किया गया है?	हाँ 1<br>ना 2	M7
महिलाओं के लिए:<br>क्या आप गर्भवती हैं?	हाँ 1<br>ना 2	M8
**कद और भार**		
मुलाकात करता की पहचान	|__|__|__|	M9
कद और भार के लिये डिवाइस	कद |__|__| भार |__|__|	M10a<br>M10b
कद	सेन्टीमीटर में (cm) |__|__|__| . |__|	M11
वजन<br>यदि स्केल के लिए बहुत बड़ा हो तब *666.6*	किलोग्राम में (kg) |__|__|__| . |__|	M12
**कमर**		
कमर के लिये डिवाइस	|__|__|	M13
कमर का घेरा	सेन्टीमीटर में (cm) |__|__|__| . |__|	M14
**Hip Circumference and Heart Rate**		
Hip Circumference	सेन्टीमीटर में (cm) |__|__|__| . |__|	M15
**Heart rate (हृदय गति)**		M16
a हर मिनट में धड़कने (पढ़ना - 1)	|__|__|__|	M16 a
b हर मिनट में धड़कने (पढ़ना - 2)	|__|__|__|	M16 b
c हर मिनट में धड़कने (पढ़ना - 3)	|__|__|__|	M16 c
**Skin Fold Thickness**		
Triceps	in (mm) |__|__|__| . |__|	MR2X
Suprailiac	in (mm) |__|__|__| . |__|	MR3X
Subscapular	in (mm) |__|__|__| . |__|	MR4X

जीव रासयनिक माप-दंड खून का ग्लूकोस		
**Blood Glucose**		
प्रश्न	उत्तर	**Code**
पिछले 12 घंटो के दौरान क्या आपने पानी के इलावा कुछ और खाया या पीया है।	हाँ 1<br>ना 2	B1
मुलाकात करता की पहचान	└─┴─┴─┘	B2
डिवाइस आई. डी	└─┴─┘	B3
खून लेने का समय	Hours : minutes └─┴─┘ : └─┴─┘<br>hrsmins	B4
सुबह की शुगर	mg/dl └─┴─┘ . └─┴─┘	B5
आज आपने कोई निर्धारित दवाई या इन्सूलिन लिया है जो कि डॉक्टर या स्वास्थ्य कर्मचारी द्वारा निर्धारित हो।	हाँ 1<br>ना 2	B6

खून का सैंपल कैल्सटरोल टराईगालिसरईडस और सीरम कगेटिन के लिये		
सेम्पल आई. डी	└┴┘	B7
कुल कैल्सटरोल	mg/dl └┴┘ . └┴┘	B8
क्या आपने पिछले 2 हफतों में बढ़े हुये कौलेस्ट्रॉल के लिये दवाई या इलाज लिया है जो कि डॉक्टर या स्वास्थ्य कर्मी द्वारा निर्धारित हो।	हाँ 1<br>ना 2	B9
Triglycerides	mg/dl └┴┘ . └┴┘	B16
Serum creatinine	mg/dl └┴┴┘ . └┘	*BX 17*

Urinary sodium, albumin and creatinine			
आपने पेशाब टैस्ट का सैंपल देने से पहले कुछ खाया तो नहीं है।	हाँ	1	B10
	ना	2	
टेक्नीशन आई. डी		└┴┴┘	B11
सेम्पल आई. डी		└┴┘	B12
दिन और समय / पेशाब टैस्ट का	Hours : minutes	└┴┘ : └┴┘<br>hrs : mins	B13
Urinary sodium	mg/dl	└┴┴┘ . └┘	B14
Urinary creatinine	mg/dl	└┴┘ . └┴┘	B15
Urinary albumin	mg/dl	└┴┴┘ . └┘	BX16
Albumin creatinine ratio	Mg/g		BX18
Serum Cystatin C	Mg/l		BX 19
Serum High Density Cholesterol	mg/dl		BX 20
Serum Low Density Cholesterol	mg/dl		BX 21

पिछले दो सप्ताहों के दौरान नीचे दी गई तकलीफों से आप आमतौर पर कितनी बार परेशान रहे है?

		बिल्कुल नहीं	कुछ दिन	आधे से ज्यादा दिन	लगभग हर रोज ही
1.	किसी काम को करने में मन न लगना या मज़ा न आना	☐	☐	☐	☐
2.	उदास, निराश या हतोत्साहित महसूस करना।	☐	☐	☐	☐
3.	नींद न आना या पूरी तरह से न सो पाना या बहुत ज़्यादा नींद आना।	☐	☐	☐	☐
4.	थकान महसूस करना या ऐसा लगना कि शरीर में ताकत नहीं है।	☐	☐	☐	☐
5.	भूख कम लगना या ज़्यादा भूख लगना ।	☐	☐	☐	☐
6.	अपने बारे में बुरा महसूस करना या महसूस करना कि आप एक असफल व्यक्ति हैं या आप को तथा आपके परिवार को आपकी वजह से नीचा देखना पड़ा है।	☐	☐	☐	☐
7.	ध्यान लगााने में परेशानी होना, जैसे कि अखबार पढ़ने या टेलीविज़न देखने में ध्यान न लगा पाना ।	☐	☐	☐	☐
8.	इतना धीरे चलना या बोलना कि दूसरे लोगों की नज़र में आ जाए या बिल्कुल इसके विपरित इतनी बेचैनी होना कि आमतौर से ज़्यादा चलना फिरना।	☐	☐	☐	☐
9.	सोचना कि मर जाएँ तो बेहतर है या खुद को किसी तरह से चोट पहुचने का मन करना।				
